# Effects of the invasion of *Ralstonia solanacearum* on soil microbial community structure in Wuhan, China

**DOI:** 10.1128/msphere.00665-23

**Published:** 2024-01-17

**Authors:** Qian-Yu Wu, Rong Ma, Xing Wang, Yi-Nan Ma, Zhi-Shan Wang, Hai-Lei Wei, Xiao-Xia Zhang

**Affiliations:** 1 State Key Laboratory of Efficient Utilization of Arid and Semi-arid Arable Land in Northern China, Key Laboratory of Microbial Resources Collection and Preservation, Ministry of Agriculture and Rural Affairs, Institute of Agricultural Resources and Regional Planning, Chinese Academy of Agricultural Sciences, Beijing, China; 2 School of Chemistry and Biological Engineering, University of Science and Technology Beijing, Beijing, China; University of Wisconsin-Madison, Madison, Wisconsin, USA

**Keywords:** *Ralstonia solanacearum*, microbial diversity, physicochemical properties, protozoa, microbial interaction networks

## Abstract

This study investigated the change in the microbiome of tomato rhizosphere soils after the invasion of *Ralstonia solanacearum* and analyzed the correlation between microbes and soil physicochemical properties. Diversity analyses of the bacteria in healthy and diseased rhizosphere soil samples (HRS and DRS) revealed that HRS had a higher species diversity and were compositionally different from DRS (*P* ≤ 0.05). Substantial differences in the relative abundance of Actinobacteria (37.52% vs 28.96%, *P* ≤ 0.05) and Proteobacteria (29.20% vs 35.59%, *P* ≤ 0.05) were identified in HRS and DRS, respectively. Taxonomic composition analysis showed ten differentially abundant genera, and seven of them (*Gaiella*, *Roseisolibacter*, *Solirubrobacter*, *Kribbella*, *Acidibacter*, *Actinomarinicola*, and *Marmoricola*) are more abundant in HRS. Soil pH and enzyme activities were negatively correlated with the abundance of *R. solanacearum*. The contents of total nitrogen (TN), total phosphorus (TP), total potassium (TK), alkaline nitrogen (alkaline N), available phosphorus (AP), available potassium (AK), NO3—N(NN), NH4^+^-N (AN), and organic matter (OM) were all significantly increased in DRS. The composition and richness of protozoa in the samples show significant differences. *Cephalobus*, *Acrobeles*, *Heteromita*, norank_Tylenchida, and *Rotylenchulus* were enriched in DRS. Microbial interaction networks revealed that the HRS networks were more complex than the DRS networks. Overall, the results of this study demonstrate that healthy soil has a more complex microbial community structure and higher enzyme activity, and the invasion of *R. solanacearum* damages the soil microbial system.

**IMPORTANCE:**

How does the invasion of *Ralstonia solanacearum* affect tomato rhizosphere bacteria and protozoa? Which microbial changes can affect the growth of *R. solanacearum*? To date, most research studies focus on bacteria, with little research on protozoa, and even less on the synergistic effects between protozoa and bacteria. Here, we analyzed the correlation between tomato rhizosphere bacterial and protozoan communities and soil physicochemical properties during the invasion of *R. solanacearum*. We found that the diversity and abundance of rhizosphere microorganisms in healthy rhizosphere soil samples (HRS) were significantly higher than those in diseased rhizosphere soil samples (DRS), and there were significant changes in soil pH and enzyme activity. Overall, in this study, the analysis of microbial changes during the invasion of *R. solanacearum* provides a theoretical basis for the prevention and control of bacterial wilt.

## INTRODUCTION

The long-term continuous cropping of tomatoes led to the outbreak of the destructive soilborne disease bacterial wilt (1). The lethal disease bacterial wilt is mainly caused by *Ralstonia solanacearum*, which can infect more than 400 important cash crops, including tomatoes, potatoes, bananas, and peppers (2, 3). *R. solanacearum* enters the plant via a root wound, migrates to the shoots through the xylem, and produces extracellular polysaccharides to block the xylem, eventually resulting in wilting and death (4). After reproduction in the plant, *R. solanacearum* moves downward and re-enters the soil through the root, becoming the source of infection for subsequent crops (5). Bacterial wilt is economically important in many crops in tropical and subtropical regions, but effective control measures are very limited. Compared to traditional physical and chemical approaches, biological agents have the characteristics of safety and sustainability. The proposal of effective biological measures for the control of soil disease depends on the complex interactions between pathogens, soil microbiota, and soil properties, which remain to be studied (6).

The invasion of *R. solanacearum* was affected by soil physical and chemical conditions and the structure of rhizosphere microbial communities (3, 7). Lee et al. (8) found the abundance of Gram-positive Actinobacteria and Firmicutes phyla was higher in healthy rhizosphere soil than in diseased rhizosphere soil, but the group of *R. solanacearum* had no changes between them. *Brevibacterium frigoritolerans* HRS1, *Bacillus niacini* HRS2, *Solibacillus silvestris* HRS3, and *Bacillus luciferensis* HRS4 had no resistance to *R. solanacearum*, but a synthetic community composed of these species has a good antagonistic effect. This means that the occurrence of soil-borne diseases is closely related to the imbalance of protective Gram-positive bacterial communities in the soil (8). The abundance of *R. solanacearum* at pH 4.90–5.60 was higher than that at pH 4.45 and pH 6.45. All bacterial communities in samples with different pH values were dominated by phyla Proteobacteria, Actinobacteriota, Acidobacteriota, and Chloroflexi, Firmicutes had the highest abundance in the samples with pH 6.45; At the genus level, *Ralstonia* was significantly enriched at pH 5.35, while *Bacillus*, *Paenibacillus*, *Pseudomonas*, and *Flavobacterium* were significantly enriched at pH 6.45. Therefore, the inhibition of soil against *R. solanacearum* was improved by alleviating soil acidification and recruiting beneficial rhizosphere bacteria (9). Bacterial wilt in tomato fields is closely related to soil nutrients and bacterial diversity. Zheng et al. reported that the soil fertility of healthy soil samples was significantly higher than that of diseased soil, in which total nitrogen (TN) content was positively correlated with *R. solanacearum*, while soil organic carbon (SOC), total phosphorus (TP), total potassium (TK), and exchangeable calcium were negatively correlated; Proteobacteria was more abundant in healthy soil, while the opposite scenario was observed for Acidobacteria. The abundance of *R. solanacearum* is positively correlated with Chloroflexi, Acidobacteria, and Planctomycetes and negatively correlated with Nitrospirae, Bacteroides, and Proteobacteria (10). Protists are an essential, yet often forgotten, component of the soil microbiome. They occupy key roles in microbial food webs as consumers of bacteria, fungi, and other small eukaryotes (11). Xiong et al. investigated the rhizosphere microbiome including bacteria, fungi, and protists of diseased and healthy tomatoes under field conditions and reached the following three conclusions: (i) the community structure of rhizosphere protozoa is a key factor in predicting plant health; (ii) phagocytic protozoa can regulate the growth of pathogenic bacteria during plant growth; (iii) the top–down regulation of rhizosphere protozoa affects plant health status (12).

The rhizosphere is the direct interface between plants and soil for material and energy exchange, and microbiomes are the most active in this area. The rhizosphere microbiome directly or indirectly promotes the healthy growth of plants by regulating nutrient utilization, secreting growth regulators, recruiting beneficial microorganisms, and inhibiting pathogenic microorganisms (13). The perturbation in the rhizosphere microbiome is directly related to plant health. Researchers found that inoculation with exogenous rhizobia in soil not only promotes the growth of soybean plants but also increases connections in rhizobacterial networks and changes the core microbes (14). Five rhizospheric Actinobacteria from wheat directly participate in soil phosphorus dissolution by secreting malic acid and phytase, thereby promoting wheat growth (15). Microbial agents mainly control bacterial wilt through three interactions: first, to inhibit the activity of metabolic enzymes of pathogenic bacteria or change the ecological conditions of plant rhizosphere soil by secreting secondary metabolites such as antibiotics and surfactants; second, to induce the plant innate immune system to respond to bacterial wilt; third, the competition between nutrition and space (16, 17). Due to the unstable application performance of a single biological control agent in the field, a beneficial microbial consortium that can better utilize existing resources and produce antibiotics may help improve the consistency and effectiveness of biological control of bacterial wilt (17). Kang et al. found that pH and soil nutrients were the main factors driving variation in the microbial community structure in an alpine wetland (18). However, further research is needed to determine whether there is a correlation between microorganisms, especially protozoa, and soil nutrients during the outbreak of bacterial wilt.

In China, 30 provinces have reported bacterial wilt, of which the incidence in the south and east is higher than that in the north and west (19). Rhizosphere microorganisms regulate the plant immune system and improve the resistance of host plants to bacterial wilt. On the contrary, plant health affects the composition of rhizosphere microorganisms, which play an important role in the fight against tomato bacterial wilt. In this study, the rhizosphere soil of healthy and diseased tomatoes collected from a net house in Wuhan was taken as samples. Based on high-throughput sequencing, the diversity of bacteria and protozoa was analyzed. At the same time, further analysis was conducted on the changes in enzyme activities and physicochemical properties of the relevant soil, and these indicators were analyzed in combination with the microbial community. By comparing the differences in microbial and physicochemical indicators between healthy and diseased rhizosphere soil samples (HRS and DRS), this study aims to explore the causes of the outbreak of *R. solanacearum* to provide a theoretical basis for the prevention and control of bacterial wilt in Wuhan.

## RESULTS

### Identification of *R. solanacearum* in soil samples

**Fig 1:**
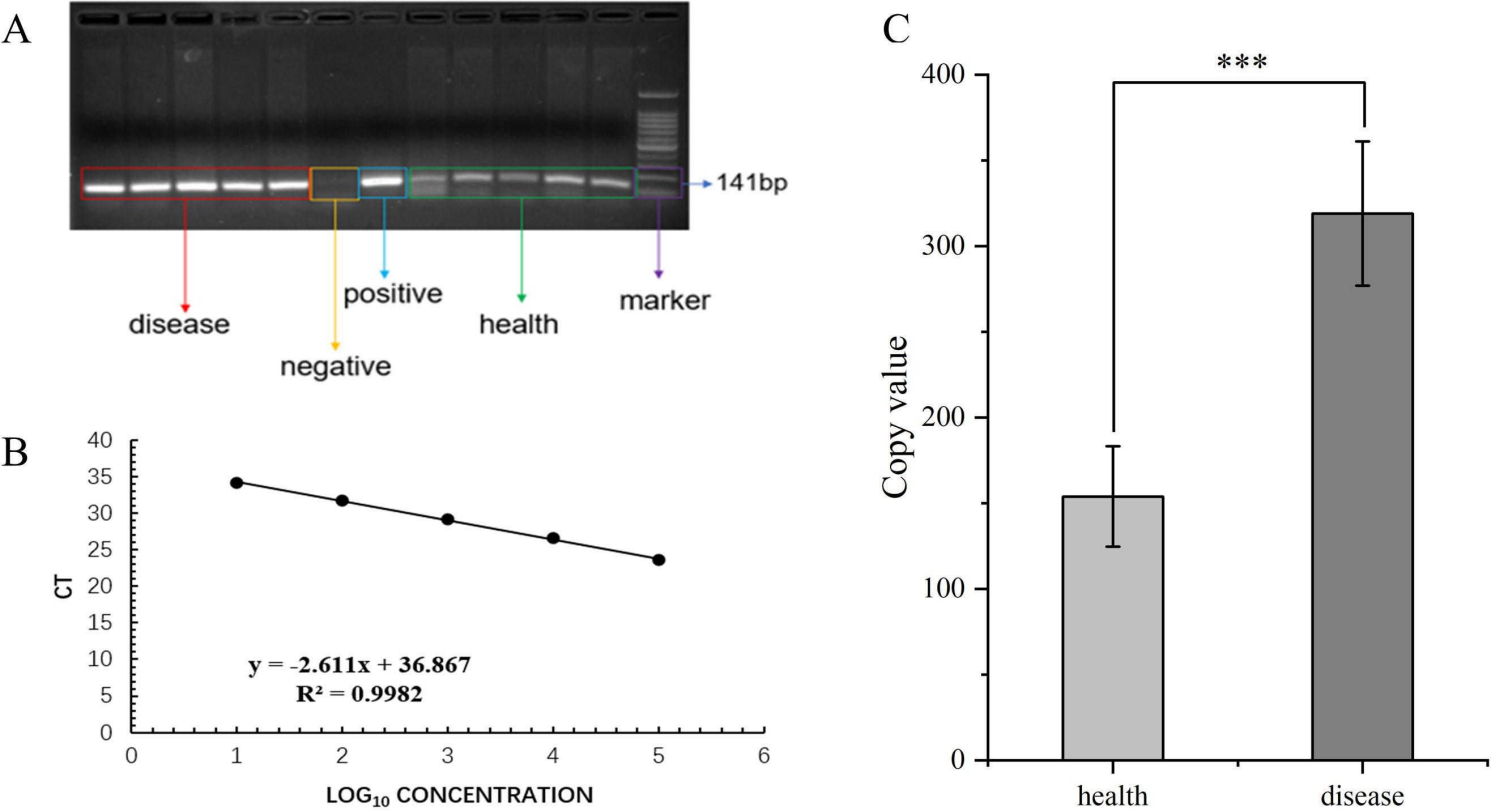
Qualitative and quantitative results of *Ralstonia solanacearum* in healthy and diseased tomato rhizosphere soils (A: qualitative results; B: standard curve; C: quantitative results).

Qualitative and quantitative detection of *R. solanacearum* in tomato rhizosphere soil samples was determined by the *fli*C gene of the flagellum subunit. Qualitative detection (Fig. 1A) showed that the amplified bands of DRS were brighter. Quantitative detection (Fig. 1C) shows that the content of *R. solanacearum* in HRS and DRS is significantly different (*P* ≤ 0.05). PCR and qPCR results showed that the content of *R. solanacearum* in DRS was significantly higher than that in HRS (*P* ≤ 0.05).

### Composition and diversity of the bacterial community in tomato rhizosphere soil

**Fig 2:**
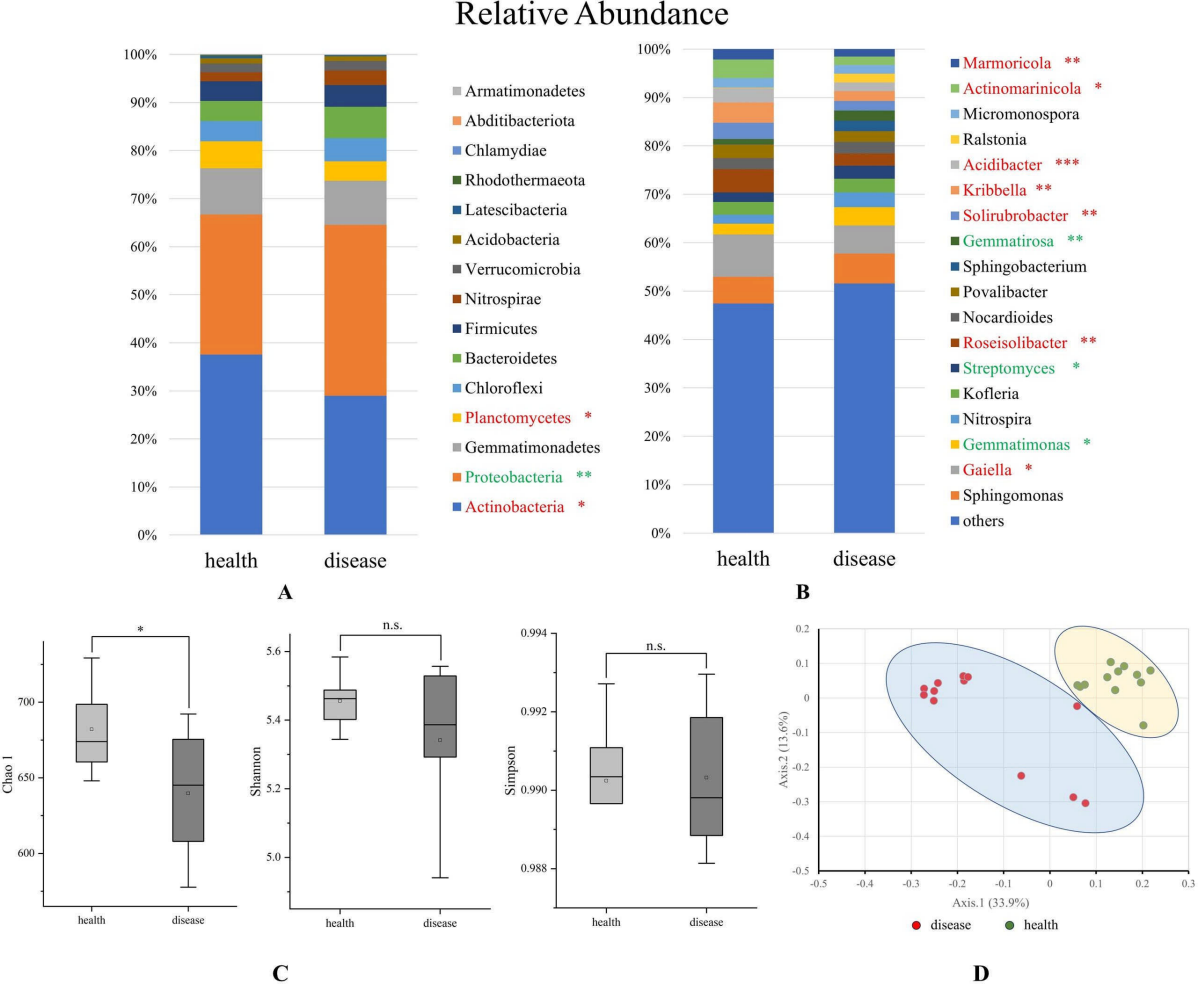
Comparison of the bacteria community structure in HRS and DRS (A: phylum level, B: genus level, C: α-diversity, D: β-diversity). Genera accounting for <1.5% of all sequences are classified into “others”. “*” represents a significant difference between the two samples. Red represents higher in HRS, and green represents higher in DRS.

Based on a 97% similarity level, the sequences were clustered to generate OTU (Operational Taxonomic Units), and the microbial groups in HRS and DRS were analyzed. All bacterial communities were dominated by phyla Proteobacteria, Actinobacteria, and Gemmatimonadetes with 28.96–37.52%, 35.59–29.20%, and 9.16–9.58% average relative abundance, respectively. Besides, the relative abundance of Planctomycetes has a significant difference (*P* ≤ 0.05) between DRS and HRS (Fig. 2A). *Gaiella*, *Roseisolibacter*, *Solirubrobacter*, *Kribbella*, *Acidibacter*, *Actinomarinicola*, and *Marmoricola* were significantly enriched at the genus level in HRS (*P* ≤ 0.05), whereas *Gemmatimonas*, *Streptomyces*, and *Gemmatirosa* increased significantly in DRS (Fig. 2B).

Chao1, Shannon, and Simpson indexes were used to reflect the *α*-diversity of the microbial community in tomato rhizosphere soils. Figure 2C showed that the microbial community richness and diversity of HRS were significantly higher than those of DRS (*P* ≤ 0.05). Based on the calculation method of Bray–Curtis distance, *β*-diversity showed that the microbial community composition was significantly different between HRS and DRS (Fig. 2D). This means that the invasion of *R. solanacearum* changed the soil bacterial community structure.

### Composition and diversity of the protist community in rhizosphere soil

**Fig 3:**
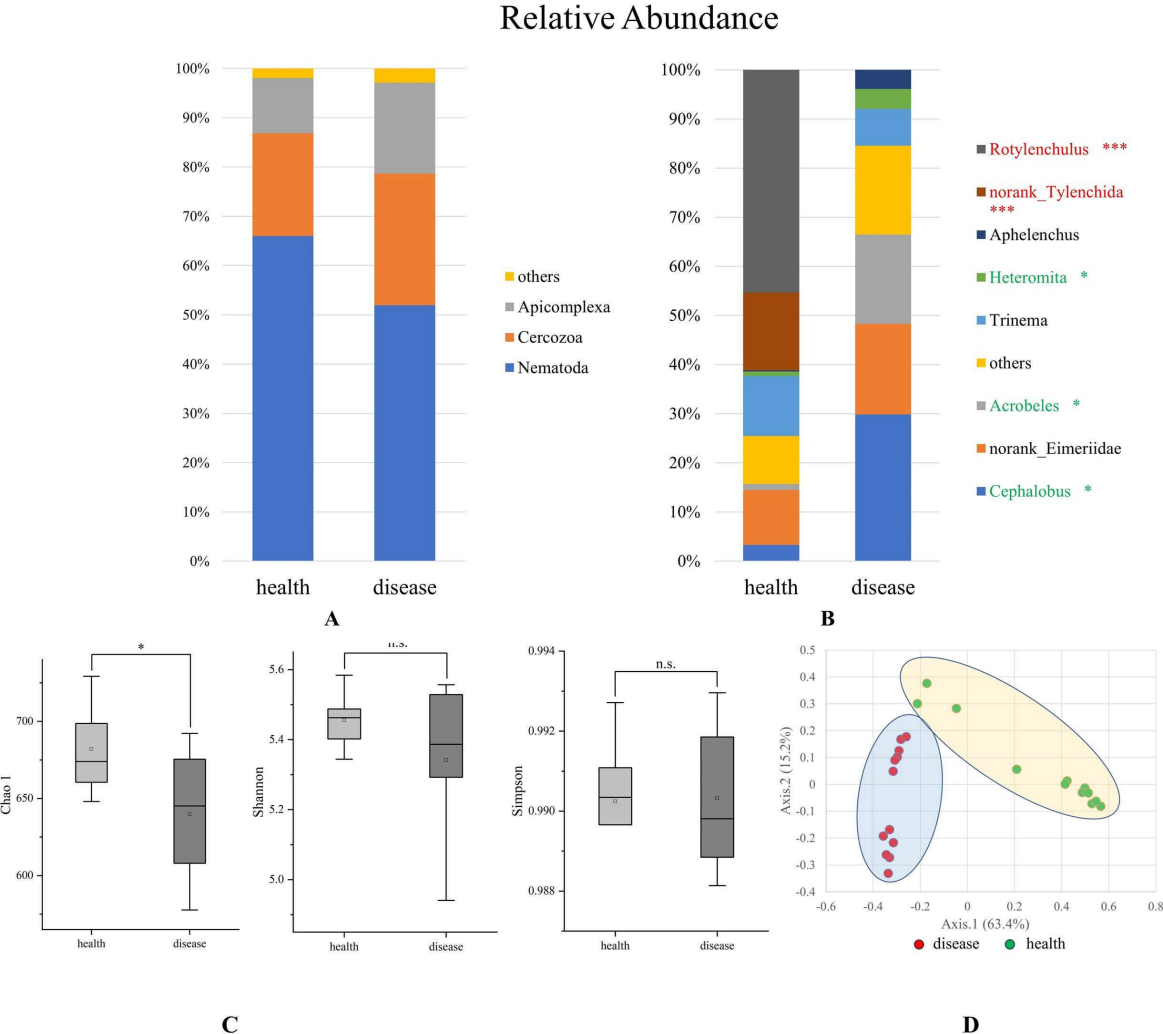
Comparison of the protist community structure in HRS and DRS (A: phylum level, B: genus level, C: α-diversity, D: β-diversity). Genera accounting for <1.5% of all sequences are classified into “others”. “*” represents a significant difference between the two samples. Red represents higher in HRS, and green represents higher in DRS.

The eukaryotic 18S rRNA gene was used to analyze rhizosphere protists, mainly protozoa and nematodes. Relative abundance analysis indicated that Apicomplexa and Cercozoa were major protozoa communities at the phylum level (Fig. 3A). Further analysis also showed that enriched communities such as *Cephalobus*, *Acrobeles*, *Heteromita*, norank_Tylenchida, and *Rotylenchulus* were associated with the invasion of *R. solanacearum* (Fig. 3B). *Cephalobus* (29.84%) and *Acrobeles* (18.17%) significantly increased in DRS, and *Rotylenchulus* (45.34%) and norank_Tylenchida (15.84%) were the main groups of HRS.

Comparative analysis of *α*-diversity indices (Chao 1, Shannon, and Simpson indexes) revealed that there was a significant difference (*P* ≤ 0.05) in the diversity of the tomato rhizosphere protist (Fig. 3C). PCoA (principal coordinates analysis) demonstrated a significant separation of the healthy and diseased tomato rhizosphere soils (Fig. 3D).

### Correlation analysis of bacteria and protist communities in HRS and DRS

**Fig 4:**
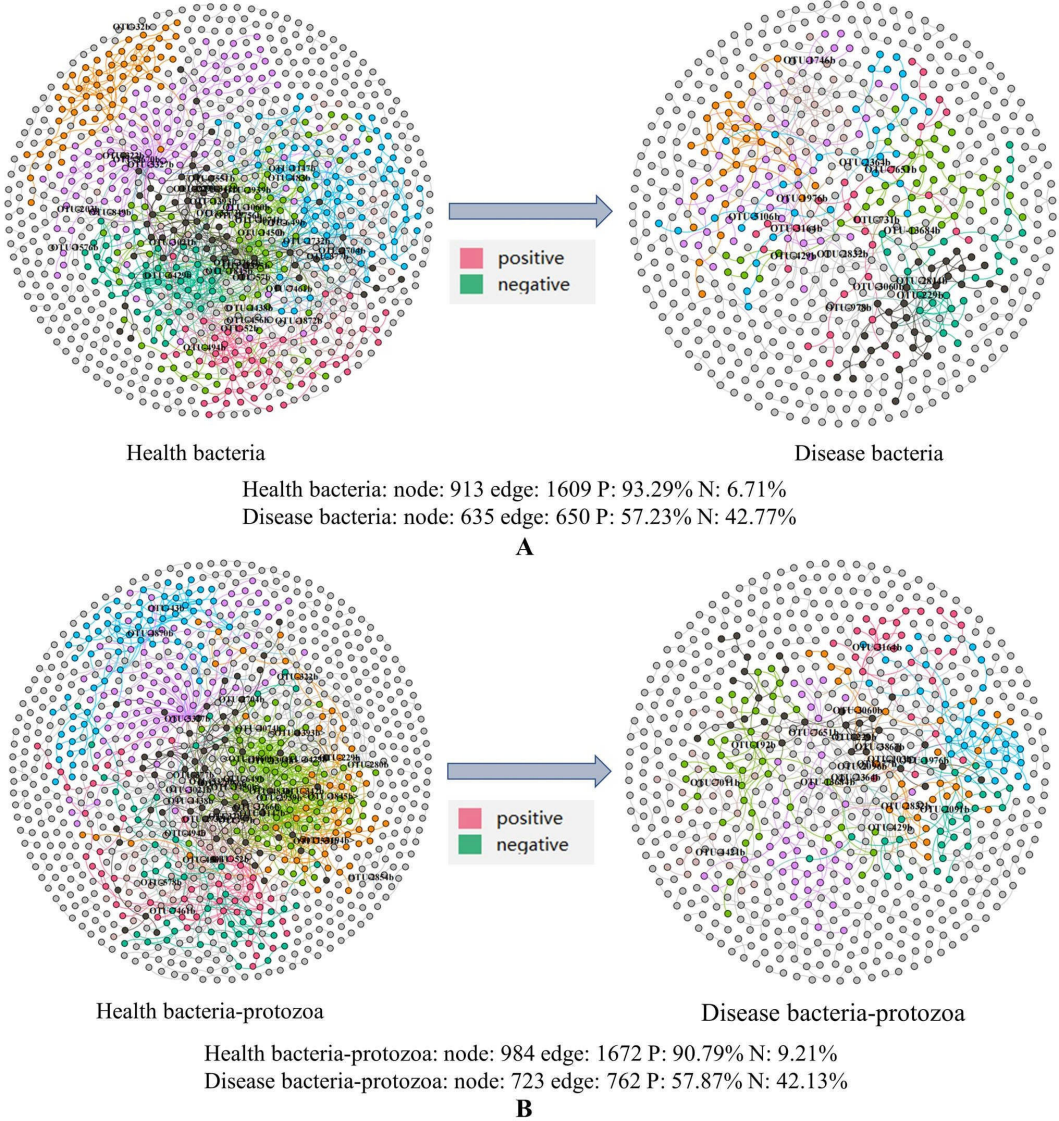
Co-occurrence network of the bacteria (**A**) and bacteria-protist (**B**) of HRS and DRS. The SparCC algorithm was used to calculate the network at the OTU level with *P* < 0.05. Each node represents a single OTU, and hub nodes are marked. The color of edges indicates the type of interaction. Red, positive; blue, negative.

Microbial interaction networks were built through the molecular ecological network analysis (MENA) pipeline and used the OTU abundance expressed as standardized relative abundances in the pipeline. The cutoff value with *P* > 0.05 and a relatively small chi-square value are selected as the threshold values. The network analysis (Fig. 4) revealed that the HRS networks contained more nodes and edges (bacteria: 913 nodes, 1,609 edges; bacteria-protist: 983 nodes, 1,672 edges) than the DRS networks (bacteria: 635 nodes, 650 edges; bacteria-protist: 723 nodes, 762 edges). Great changes have taken place in microbial interactions. The proportion of HRS-positive interactions (bacteria: 93.29%, bacteria-protist: 90.79%) is higher than that of DRS (bacteria: 57.23%, bacteria-protist: 57.87%). The module connectivity (Zi >2.5) and among-module connectivity (Pi >0.62) values were measured as core hubs in the rhizosphere soil of HRS and DRS (20). A total of 72 core hubs were obtained in the HRS network (bacteria: 35, bacteria-protist: 37), while 31 core nodes were obtained in the DRS network (bacteria: 14, bacteria-protist: 17). The core hubs of each network are different, except for OTU 229b, which belonged to *Sphingomonas*. Network complexities, as measured by the node number and the average connectivity of the nodes (21), were considerably different between HRS and DRS. Whether it is for bacteria or bacteria-protist, the HRS network was more complex than the DRS network. The network analysis results showed that the invasion of *R. solanacearum* has altered the interaction of microorganisms.

### Effects of abiotic factors on the tomato rhizosphere microbial community

**Fig 5:**
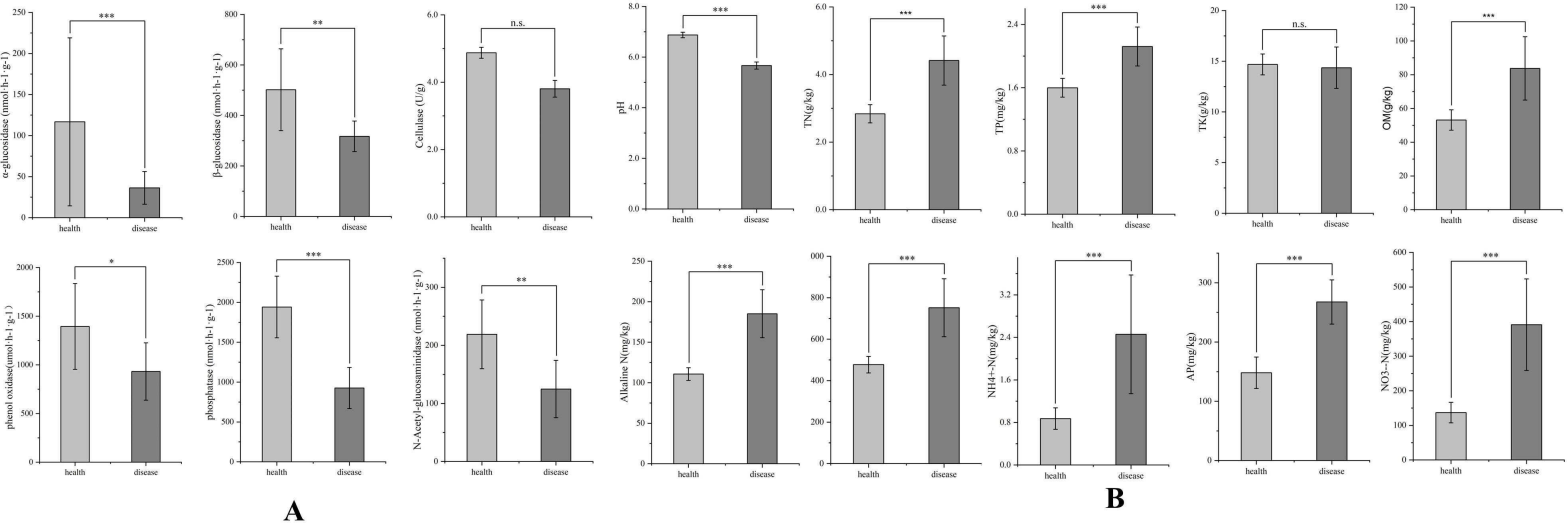
Enzyme activity (**A**) and physicochemical properties (**B**) in healthy and diseased tomato rhizosphere soils. “*” represents a significant difference between the two samples. TN: contents of total nitrogen, TP: total phosphorus; TK: total potassium; alkaline N: alkaline nitrogen; AP: available phosphorus; AK: available potassium; AN: NO_3_^—^N, NN: NH_4_^+^-N; OM: organic matter.

Compared with the DRS, the activities of *α*-glucosidase (AG), *β*-glucosidase (BG), phenol oxidase (PPO), phosphatase (AP), and N-acetyl-glucosaminidase (NAG) significantly increased in the HRS, and there was no difference in *β*-cellobiosidase (CB) activities between the two samples (Fig. 5A). The enzyme activity in the HRS is significantly higher than that in the DRS (*P* ≤ 0.05). The physicochemical properties of the two tomato soil samples were significantly different, except for total potassium (TK) (*P* ≤ 0.05), and the nutrient content of DRS is higher than that of HRS, while the opposite scenario holds for pH (Fig. 5B).

**Fig 6:**
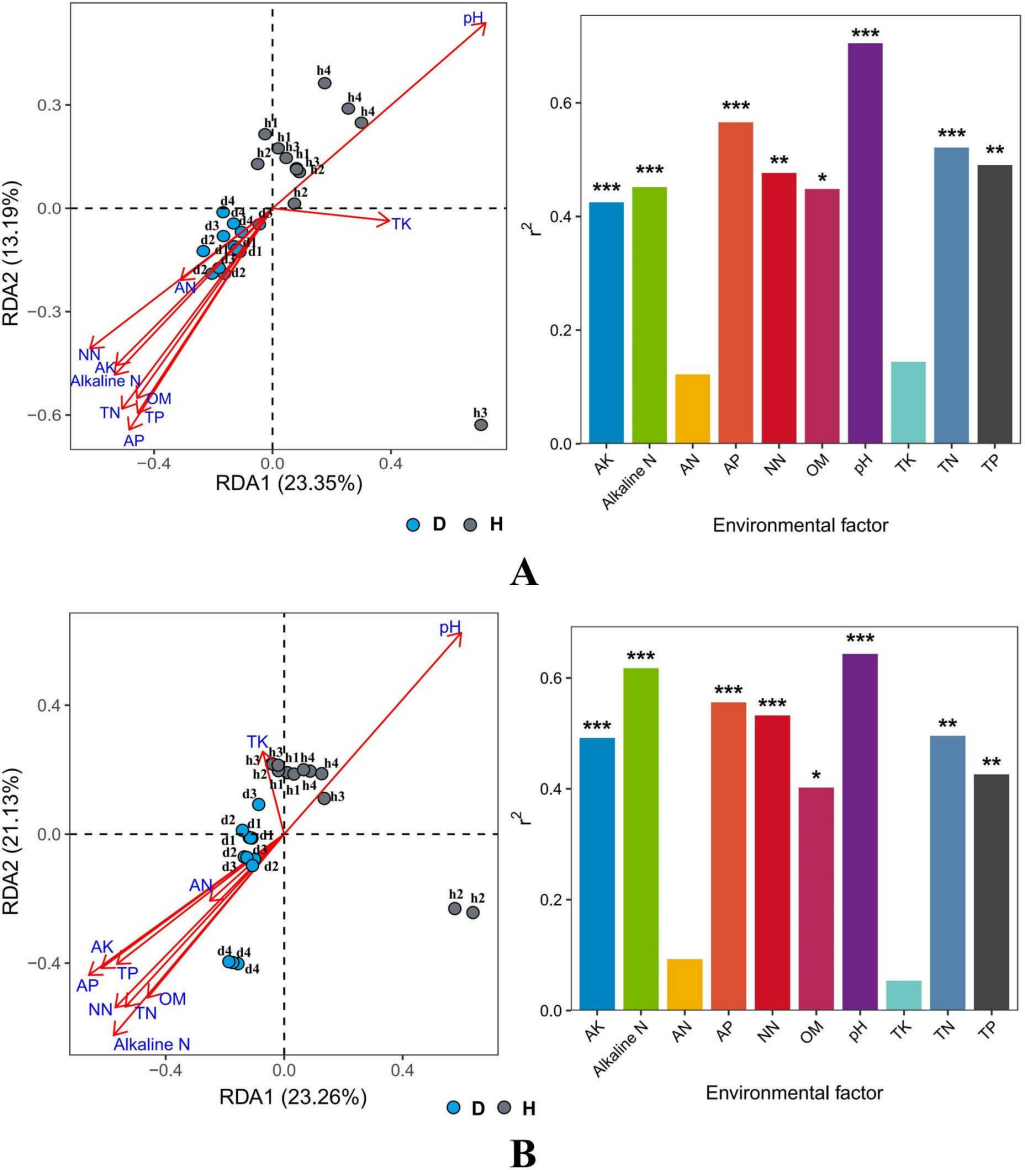
The redundancy analysis (RDA) ordination plot (A: bacteria, B: protozoa) showed the relationship between the relative abundance of microbial and soil physicochemical parameters and enzyme activities in the tested samples (D: diseased, H: healthy). “*” represents a significant difference between the two samples. TN: contents of total nitrogen; TP: total phosphorus; TK: total potassium; alkaline N: alkaline nitrogen; AP: available phosphorus; AK: available potassium; AN: NO_3_^—^N, NN: NH_4_^+^-N; OM: organic matter.

The relationship between microbial community structure (bacteria and protozoa) and soil properties was analyzed by redundancy analysis (RDA). According to Fig. 6, available potassium (AK), alkaline nitrogen (alkaline N), available phosphorus (AP), pH, TN, NN, and OM were correlated with both bacteria and protozoa communities; Between them, the correlation between TN and bacteria is more pronounced, while the correlation between NN and protozoa is more pronounced (*P* ≤ 0.05), and AN and TK are completely unrelated.

## DISCUSSION

This study aimed to test whether the invasion of *R. solanacearum* is influenced by soil properties and can alter the structure of soil microbial communities. The plant rhizosphere acts as the first line of defense against the invasion of pathogens, and the rhizosphere microbiome is directly related to plant health and disease development (13). qPCR of the *fli*C gene showed that the abundance of *R. solanacearum* in DRS was significantly higher than that of HRS in this study. High-throughput sequencing in this study and other research indicated the same trend (22). In addition, this study determined the relative abundance and diversity of the microbiome in healthy and diseased rhizosphere soils, detected relevant soil chemistry properties, and analyzed microbial interactions based on MENA. Higher pathogen abundance decreased the *α*-diversity of the rhizosphere bacterial community as well as connections in co-occurrence networks (22).

Based on the result of high-throughput sequencing at the phylum level, HRS had a relatively higher abundance of Actinobacteria and Planctomycetes and a lower abundance of Proteobacteria than DRS. It has been reported that Proteobacteria is involved in the prevention of *Rhizoctonia solani* in soil (23). Zheng et al. (24) reported that the abundances of Actinobacteria and Gemmatimonadetes declined when bacterial wilt disease occurred (24). The 16S rRNA gene of the Gemmatimonadetes was frequently and abundantly detected in various terrestrial environments, which is currently recognized as one of the dominant soil phyla (25). Although there was no significant difference in Gemmatimonadetes at the phylum level in this study, the results were completely different at the genus level. In addition, it is reported that Chloroflexi could be a potential disease-inducing phylum and was positively correlated with tobacco bacterial wilt disease rate (26), and the opposite scenario holds for Firmicutes. However, the results of this study do not reflect the same trend. Previous studies showed that the taxonomic, structural, and functional composition of soil microbial communities changed when bacterial wilt occurs in the soil (7). Similarly, DRS exhibited a higher microbial diversity than DRS in this study, especially beneficial microbes (e.g., *Gaiella* and *Solirubrobacter*) that can control soil-borne diseases, improve soil nutrients, and promote plant growth. For example, the genus *Gaiella* is known as an important organic matter decomposer and is involved in carbon cycling, and the genus *Solirubrobacter* could promote the metabolism rate of plant growth (27); Thereby, it can be inferred that these beneficial groups are positively correlated with soil quality and plant health.

Compared to DRS, the biological and enzyme activity of HRS was significantly increased. Previous studies showed that the abundance of bacteria is influenced by the acidity of the soil (9). The imbalance of the bacterial community caused by excessively acidic soil is one of the main causes of soil-borne diseases (7, 24, 28). Zhang et al. found that the occurrence of bacterial wilt and the abundance of *R. solanacearum* significantly reduced when regulating soil pH from 5.45 to 6.0; meanwhile, a slightly acidic level could promote the enrichment of beneficial bacteria to suppress bacterial wilt (9). In this study, the pH of HRS was between 6.8 and 7.0, while that of DRS was between 5.5 and 5.8. Soil enzymes catalyze several biochemical reactions and are widely used for evaluating soil quality (7). They are closely associated with the C (glucosidase), N (urease), and P (phosphatase) cycle (28). Wang et al. (29) found a significantly negative correlation between *β*-glucosidase activity and soil pH (29). The synthesis, release, and stability of phosphatase were also affected by soil pH (30). Similarly, this study found that the enzyme activities were negatively correlated with soil pH, and HRS enzyme activities were significantly higher than those in DRS. An acidic environment can reduce soil enzyme activity. Soil enzyme activities regulate nutrient fluxes and promote plant growth. Nutrients are important for the growth and development of plants and microbes, and nutrient deficiencies in soils weaken plants, making them more vulnerable to diseases (18). Many studies have shown that soil pH and SOC, TN, TP, and TK content are negatively correlated with the soil-borne pathogen population and severity of diseases (7, 9, 20, 28). Higher nutrient content enhances the growth of plants and inhibits their pathogens (9, 18). However, in this study, the healthy plant rhizosphere soils had lower TN, TP, AN, OM, AK, AP, NO_3_^-^-N, and NH_4_^+^-N content than the diseased plant rhizosphere soils, except TK, which are completely opposite to those before. The possible reason is that the outbreak of bacterial wilt affects the nutrient intake of plants and leads to the residue of soil nutrients.

As an important microbial group in the rhizosphere microbiota, protists have received less attention (11). Protists have many functions in the rhizosphere: some prey on bacteria and fungi as rhizosphere consumers in the soil food web, some are parasitic on plants, and some also participate in the process of decomposing organic matter and play an important role in the carbon and nitrogen cycle and nutrient transformation (31, 32). This study sequenced the 18S rDNA amplified fragment and only selected protozoa and nematodes as targets. Soil nematodes have long served as bioindicators, and the ecological indices of nematode communities reveal natural and human-induced changes in soil ecosystems (33). The invasion of *R. solanacearum* in DRS significantly reduced the number of soil nematodes. Predatory protozoa are a key component of soil biodiversity involved in soil fertility and plant productivity, and their contribution to ecosystem functions is regulated by soil chemical components (11). Through their predatory activity, protists release nutrients from their prey’s biomass, making them available to plants and other organisms in their environment, while stimulating the rate of soil organic matter decomposition (34). The protozoa at the phylum level mainly include Cercozoa and Apicomplexa. As important predators and decomposers, the ecological roles of protozoa in belowground food webs and soil biogeochemical processes are of great significance (31). Apicomplexa has a higher abundance in the diseased sample, which may be related to pH (32). Another interesting point in this study is that *Rotylenchulus* and norank-Tylenchida, which have significant abundance advantages in healthy soil samples, are both plant parasites. *Heteromita*, *Arcobeles*, and *Cephalohus*, as the dominant groups in the diseased samples, are all bacterial-feeding protozoa. That is to say, the protozoa were mainly dominated by plant parasites in HRS, while in the DRS, they were mainly bacterial-feeding protozoa, which may be related to the change of the root state of plants during the invasion of *R. solanacearum*.

Based on microbial network analysis, it was found that the HRS networks were more complex and contained more interacting microbial species than the DRS networks. In the HRS network, different OTUs were tightly connected and formed a more stable network, which is likely related to the health of plants and soil (35). Besides, the HRS networks had more core nodes than the DRS networks. More core nodes made the HRS networks more stable and ordered than the DRS networks. More interactions among microbial communities help soil bacteria fulfill functions such as participating in nutrient cycling, promoting plant growth, and suppressing pathogens (36). More interacting bacteria in the HRS network also means more exchange of metabolites and information among microbial species, which makes the HRS networks work more efficiently than the DRS networks. OTU-229 is a common core hub among the four networks, and there were no protozoan groups present in thir core hubs, which indicates that the main changes in soil microbial groups during the invasion of *R. solanacearum* were caused by bacteria (37). Hence, the microbial networks of HRS could well-explain the lower incidence of pathogens such as *R. solanacearum*. In contrast to the HRS networks, microbial populations of the HRS networks were connected by fewer edges (interactions), and thus the exchange of materials and information between microbial species was possibly hampered and decreased. The better-organized microbial networks of HRS may well-suppress bacterial wilt disease, while the unstable microbial network of DRS could lead to bacterial wilt disease.

### Conclusion

The results of this study suggest that the soil microbial community and properties were altered during the occurrence of bacterial wilt. HRS had a higher diversity in microbial community diversity and enzyme activity but a lower nutrient content. Protists are also an important component of soil microorganisms, and nematodes exhibit a higher abundance than protozoa. Microbial interaction networks analysis illustrated that the HRS networks were more complex than the DRS networks, and the invasion of *R. solanacearum* affected the composition and interaction of bacterial communities. In conclusion, we found that a complex microbial community structure, with a higher pH and lower soil nutrients, can inhibit the outbreak of bacterial wilt, and these findings laid the foundation for the prevention of *R. solanacearum* in the Wuhan area.

## MATERIALS AND METHODS

### Collection and DNA extraction of soil samples

Tomato rhizosphere soil was collected from a net house in the East–West Lake District, Wuhan, China (30.62 N, 114.14 E). HRS and DRS samples were collected separately from four different points in the experimental area. Soil samples were divided into several parts; those used for DNA extraction were stored in the refrigerator at –80°C; hose used for soil enzyme activity determination shall be stored at –20°C; those used to determine the physical and chemical properties shall be placed in a ventilated place for drying and sieved with 2-mm, 1-mm, and 0.15-mm sieves, respectively.

### Determination of *R. solanacearum* in soil

The *fli*C gene of the flagellum subunit was amplified to evaluate *R. solanacearum*. The forward primer (5′-GAACGCCAACGGTGCGAACT-3′) and reverse primer (5′-GGCGGCCTTCAGGGAGGTC-3′) were used in PCR and qPCR reactions (38).

### High-throughput sequencing and data analysis

The FastDNA Spin Kit For Soil (MP) kit was used to extract DNA. Then, DNA was quantified using a NanoDrop One Spectrophotometer (Thermo Scientific, USA) and stored at −80°C until further analysis. The bacterial 16S rRNA V3–V4 region and protist 18SV4 region were amplified. Bacterial primers: 341F: (5′-CCTACGGGNGGCWGCAG-3′), 805R: (5′-GACTACHVGGGTATCTAATCC-3′) (39). Protist primers: 18SV4F: (5′-GGCAAGTCTGGTGCCAG-3′), 18SV4R: (5′-ACGGTATCTRATCRTCTTCG-3′) (40). The amplified products were examined using 1.0% (v/v) agarose gel electrophoresis and sent to Bioengineering (Shanghai) Co., Ltd. for sequencing. The sequencing platform was Miseq 2 × 300 bp, with an average of 40,000 sequencing data.

These sequences were demultiplexed and quality-filtered using Vsearch (2.7.2) on the Galaxy platform (41). Cluster the sequences according to 97% similarity and then download the OTU table and sequence file; compared with the database [16S: RDP (rdp_16 s_v16_sp) and 18S (SILVA database 138.1Version)] and delete nonbacterial DNA (mitochondria, chloroplasts, fungi, and archaea) sequences in OTU table and FASTA sequence files; conduct downstream analysis after reannotation. The molecular ecological network is constructed based on the RMT (Random Matrix Theory) method using the MENA platform (http://ieg4.rccc.ou.edu/MENA/) (42). Utilize the online platform MicrobiomeAnalysis (https://www.microbiomeanalyst.ca/). Clustering and correlation: the pattern search tool is used to analyze related groups of pathogenic bacteria (43). Origin Pro2021 and SAS 9.4 software are used for data statistics and analysis, respectively, and the online platform ImageGP (http://www.ehbio.com/ImageG/) is used for visual operation (44).

The raw high-throughput sequencing data were submitted to the NCBI database under the BioProject numbers PRJNA955316 and PRJNA955455.

### Analysis of soil enzyme activity

Soil enzyme activity was detected by the fluorescence microplate assay method. After hydrolysis of standard substrates (MUB) of different enzymes, 4-methylumbelliferyl or 7-amino-4-methyl coumarin are produced (24). The activity of soil extracellular enzymes is characterized by measuring the intensity of their fluorescence values. The enzyme to be tested and its corresponding substrate are shown in Table 1.

**TABLE 1:** Extracellular enzymes and corresponding substrate

Enzyme	Substrate
Phosphatase (AP)	4-MUB-phosphate
*α*-Glucosidase (AG)	4-MUB-α-d-glucoside
*β*-Glucosidase (BG)	4-MUB-*β*-d-glucoside
N-Acetyl-glucosaminidase (NAG)	4-MUB-N-acetyl-*β*-d-glucosaminide
Phenol oxidase (PPO)	l-DOPA
*β*-Cellobiosidase (CB)	4-MUB-*β*-d-cellobioside

The steps are as follows: (i) The sample was put into a 4°C refrigerator for thawing the day before the enzyme activity was measured and then sieved through a 2-mm sieve. (ii) Determination of soil moisture: weigh 10 g of the sample and put it into an aluminum box. Dry at 105°C for 8 hours to constant weight. (iii) Determination of soil pH as the reference standard for adjusting buffer pH. (iv) Reagent preparation: the final concentration of MUB is 10 µM; the final concentration of 4-MUB-phosphate, 4-MUB-α-d-glucoside, 4-MUB-*β*-d-glucoside, 4-MUB-N-acetyl-*β*-d-glucosaminide, and 4-MUB-*β*-d-cellobioside is 200 µM. The final concentration of l-DOPA is 25 mM; 50 mM sodium acetate buffer; 1M sodium hydroxide solution. (v) Weigh 1 g of fresh soil and put it into a 500-mL beaker. Add 100 mL of sodium acetate buffer solution and mix well; use a magnetic stirrer to stir for 1–2 min, quickly pour it into the solution container, and add it into the corresponding microporous plate within 20 min (except for the white ELISA plate for l-DOPA, the black ELISA plate is used for others). Reagents must be added to the ELISA plate in strict accordance with the volume and order. The specific combinations are sample fluorescence (200 µL sample suspension +50 µL substrate), standard fluorescence (200 µL buffer +50 µL standard solution), standard control (200 µL sample suspension +50 µL standard solution), sample control (200 µL sample suspension +50 µL buffer), and substrate control (200 µL buffer +50 µL Substrate). After sealing the ELISA plate, culture it in the dark for 4 hours (20–24 hours for l-DOPA). Before the final determination, 10 µL NaOH (1 mM) was added to terminate the reaction. The fluorescence was measured with a multifunctional enzyme marker (Scientific Fluoroskan Ascent FL, Thermo) at the excitation wavelength of 365 nm and the emission wavelength of 450 nm (l-DOPA is measured at 460 nm with absorbed light).

### Analysis of soil physicochemical properties

Soil pH and contents of TN, TP, TK, alkaline N, AP, AK, NO_3_^-^-N(AN), NH_4_^+^-N (NN), and OM were determined as previously described (28).
